# Microstructure and Mechanical Properties of Friction Stir Welded 2205 Duplex Stainless Steel Butt Joints

**DOI:** 10.3390/ma14216640

**Published:** 2021-11-04

**Authors:** Mohamed M. Z. Ahmed, Khalil Hajlaoui, Mohamed M. El-Sayed Seleman, Mahmoud F. Elkady, Sabbah Ataya, Fahamsyah H. Latief, Mohamed I. A. Habba

**Affiliations:** 1 Mechanical Engineering Department, College of Engineering at Al Kharj, Prince Sattam Bin Abdulaziz University, Al Kharj 16273, Saudi Arabia; moh.ahmed@psau.edu.sa; 2 Department of Mechanical Engineering, College of Engineering, Imam Mohammad Ibn Saud Islamic University, Riyadh 11432, Saudi Arabia; smataya@imamu.edu.sa (S.A.); fhlatief@imamu.edu.sa (F.H.L.); 3 Department of Metallurgical and Materials Engineering, Faculty of Petroleum and Mining Engineering, Suez University, Suez 43512, Egypt; mohamed.elnagar@suezuniv.edu.eg (M.M.E.-S.S.); mfathykady90@gmail.com (M.F.E.); 4 Mechanical Department, Faculty of Technology & Education, Suez University, Suez 43518, Egypt; Mohamed.Atia@suezuniv.edu.eg

**Keywords:** 2205 DSS, friction stir welding, FSW, microstructures, hardness, tensile properties, fracture surfaces

## Abstract

Friction stir welding (FSW) as a solid-state process is an excellent candidate for high softening temperature materials welding; however, extending the tool life is required to make the process cost-effective. This work investigates the use of a high pin to shoulder ratio (65%) tungsten carbide (WC) tool for friction stir welding of 5 mm thick 2205 DSS to extend the tool life of this low-cost tool material. In addition, the effect of FSW parameters in terms of rotational rates, travel speeds, and downward forces on the microstructural features and mechanical properties of the welded joints were investigated. Characterization in terms of visual inspection, macro and microstructures, hardness, and tensile testing was conducted. The obtained results indicated that the combined rotational rate, travel speed, and downward force parameters govern the production of defect-free joints. The 2205 DSS friction stir welds show an enhancement in hardness compared to the base material. The stir zone showed a significantly refined grain structure of ferrite and austenite with the reduction in the average grain size from 8.8 µm and 13.3 µm for the base material to 2.71 µm and 2.24 µm, respectively. Moreover, this joint showed higher yield strength and ultimate tensile strength compared to the DSS as-received material.

## 1. Introduction

It is well established that 2205 duplex stainless steel (2205 DSS) has almost equal austenitic (γ-phase) and ferritic (α-phase) phases. It is increasingly used in many engineering applications as an alternative to austenitic and ferritic stainless steel because of its good combination of high strength and corrosion resistance [1,2,3]. The 2205 DSS has been extensively used in the petrochemical and marine industries [4,5]. The obtained α-phase to the γ-phase ratio in the DSS microstructures is mainly related to the ratio of the stabilizing alloying elements for both α and γ phases, in addition to its production method and applied heat treatment program [6,7]. Heat treatment is an effective technique to improve the hardness properties of different heat-treatable ferrous and nonferrous alloys. Moreover, 2205 DSS is a non-heat-treatable alloy. Thus, precisely controlled thermomechanical treatment and plastic deformation are required to improve the surface and/or bulk mechanical properties [8,9]. The microstructure of 2205 DSS permits unique mixtures of α-phase and γ-phase chemical and mechanical properties. The α-phase allows high strength besides corrosion resistance, whereas the austenite influences ductility [10,11]. The microstructural changes of the 2205 DSS welded joints using the fusion welding technique showed undesirable features due to the composition of filling rod materials and temperature variation during the thermal welding cycle [1,12,13]. This leads to a loss in mechanical properties and corrosion resistance. Generally, the thermal cycle during fusion welding may also lead to solidification cracking in the welded zone and coarsening in the grain structure of the heat-affected zone (HAZ) [14]. Furthermore, the lower productivity of the arc welding techniques limited their applications in welding DSS parts. To avoid these difficulties, precise control of the weld chemical composition and working temperature are often recommended during the welding process. As a solid-state technique, friction stir welding (FSW) facilities [15,16] have been developed and used extensively to weld ferrous [17,18,19] and nonferrous [20,21,22] alloys in similar [19,23,24] and dissimilar [25,26,27] joints in various industrial applications. The FSW technique is recommended to join 2205 DSS. Sarlak et al. [28] examined the corrosion behavior and mechanical properties of the friction stir welded (FSWed) joints of 1.5 mm DSS sheet performed at a rotational speed of 800 rpm and various welding speeds from 50–150 mm/min using a WC-based tool. They remarked decreasing in grain size of the stir zone (SZ) for both α and γ phases with an increase in the welding speed. Wang et al. [29] faced serious sticking problems in FSW of 1.86 mm 2205 DSS with tungsten–rhenium-based FSW tool at a constant travel speed of 100 mm/min with the range from 300 to 600 rpm rotational welding speeds. Incomplete penetration was detected at 300 rpm, and a groove-like defect was attained at the highest rotation speed of 600 rpm. Moreover, the sound joints were achieved at rotational speeds from 350 to 500 rpm. Sato et al. [7] investigate the effect of FSW on the mechanical properties and microstructure of 2507 super duplex stainless steel (SDSS) welded at 450 rpm and 1 mm/min using a polycrystalline cubic boron nitride tool. They concluded grain refining achievements of both the γ and α phases in the SZ through the dynamic recrystallization. Saeid et al. [30] investigated the mechanical properties and microstructure for the bead-on-plate welding of 2 mm thickness 2205 DSS at a rotational speed of 600 rpm and various travel speeds from 50 to 250 mm/min. Sound 2205 DSS joints were produced at travel speeds in the range of 50–200 mm/min. Whereas, a tunnel was detected of the joint welded at 250 mm/min. They also concluded that the microstructures features and mechanical properties are related to the processing parameters. Recently, Ahmed et al. [1] investigated the applicability of FSW as a new welding method to groove filling 2205 DSS instead of the gas tungsten arc welding (GTAW) technique. They reported that the yield strength (YS), ultimate tensile strength (UTS), and elongation (E%) of the FSWed joints are enhanced over the GTAW joints by 21%, 41%, and 66%, respectively. The tool design and material are important parameters to achieve sound joint in FSW [22,31]. As mentioned in the above brief literature, many studies succeeded in friction stir welding of DSS at the thickNess less than 5 mm; however, still FSW of this material due to the increasing demand in the petroleum industry at a thickness of 5 mm or higher is a challenge in selecting processing parameters and tool material and design. Thus, the current work improves the knowledge on the relationship between the joint quality of the DSS 2205 friction stir welds and the combined factors of rotation speed, welding speed, and applied downward force. The effect of FSW parameters, in terms of rotational speed travel speed and downward force on the microstructure and mechanical properties of the 5 mm sheet 2205 DSS using tungsten carbide (WC) tool in a one-pass welding process to produce 2205 DSS butt-joints, is discussed.

## 2. Methodology

**Figure 1:**
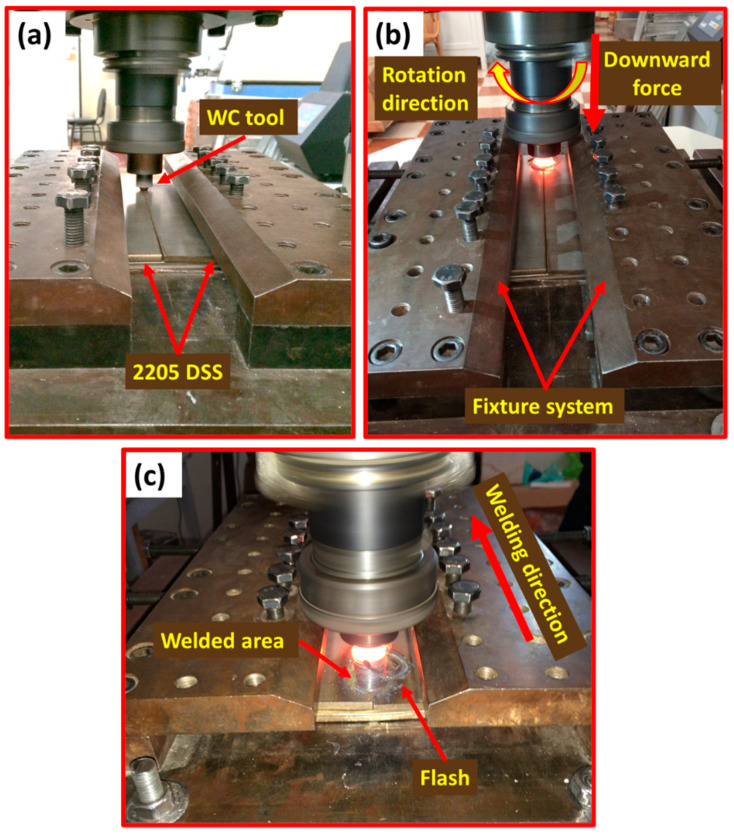
(**a**,**b**) front photos illustrate the FSW facilities and fixture system to weld 2205 DSS but joins. (**c**) back photo shows starting the welding process and direction.

**Figure 2:**
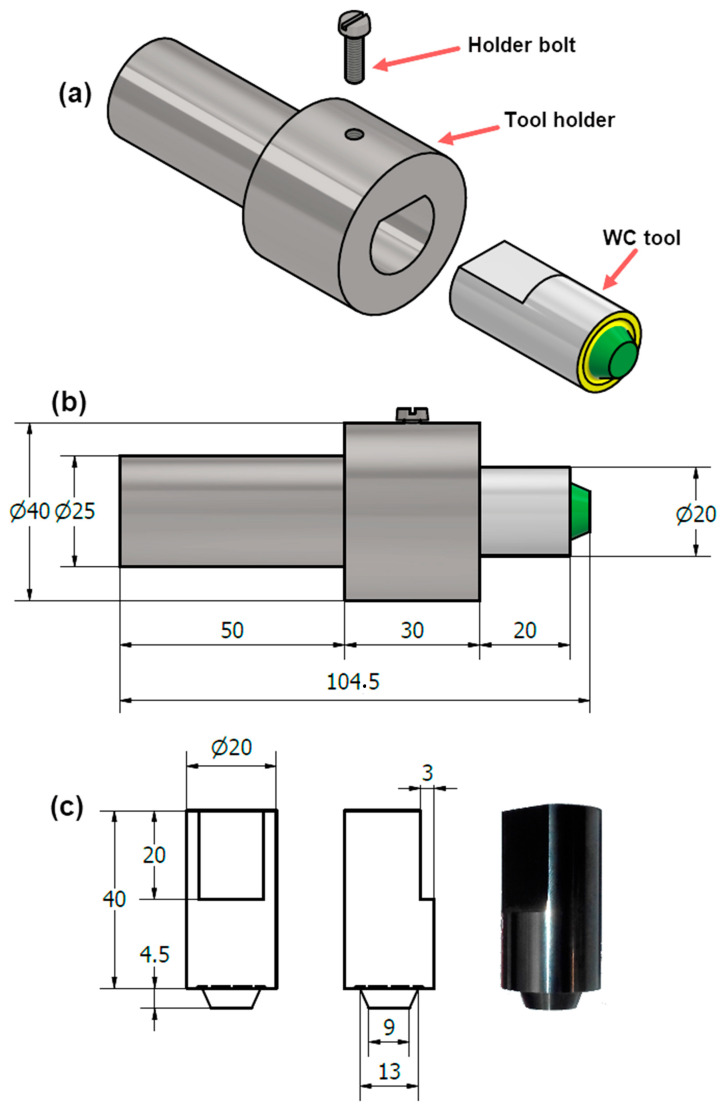
(**a**) Exploded drawing FSW tool parts (**b**) dimension of FSW tool (**c**) real view and dimensions of WC tool geometry (unit: mm).

The initial material was a hot rolled 2205 DSS sheet with a size of 1000 mm × 2000 mm × 5 mm. The chemical composition of the DSS base metal (BM) obtained using Foundry-Master pro, (Oxford Instruments, Abingdon, United Kingdom) is listed in Table 1. The BM sheet was cut into plates of 200 mm× 100 mm × 5 mm and then subjected to FSW in butt weld configurations. Welding was conducted using a full automatic Egyptian welding machine (EG-FSW-M1) (Suez University, Suez, Egypt) [17,26], as seen in Figure 1. This was performed at various welding parameters of rotational speeds, travel speeds, and downward forces, as given in Table 2. The 2205 DSS friction stir welds (FSWs) were performed in the rolling direction. The used FSW tool was designed and made of a WC-based material. Figure 2a,b show the exploded and the assembly drawings of the WC tool and its holder, respectively. Furthermore, Figure 2c illustrates the multiview of the WC tool and its photograph. Furthermore, Table 3 lists the shoulder and pin dimensions. The tool was tilted 3° from the normal plate direction for all the friction stir butt welds.

A visual test and macro examination of the welded joints were carried out to discover the welding defects on the weld surfaces and across 2205 DSS butt welded joints. Macro and metallographic specimens were cut perpendicular to the path FSW direction and ground with SiC abrasive papers up 2400 grit, followed by polishing to 0.05% alumina surface finish. The polished samples were etched using a solution of 5 g CuCl_2_ in 100 mL of HCl and 100 mL of ethanol for 25 s. The microstructural investigation was carried out using the optical microscope (Zeiss Axiovert 25 CA Inverted Reflected Light Microscope, Oberkochen, Germany) and Quanta FEG 250 scanning electron microscopy (SEM) (FEI Company, Hillsboro, OR, USA) equipped with an AMETEK energy-dispersive spectroscope (EDS). The grain size measurements of FSWed and the BM were measured using the grain interception method via Olympus Stream Motion. The hardness measurements of the produced welds were taken on the cross-sections perpendicular to the welding path direction at the center of the joint thickness with a Vickers Hardness Tester machine type HWDV-75 (TTS Unlimited, Osaka, Japan) using an applied load of 20 N and a holding time of 20 s. Tensile specimens were extracted perpendicular to the welding direction and prepared according to ASTM E8/E8M-16a. The dimensions of the tensile test specimen were 5 mm thickNess, 12.5 mm width, and 50 mm gauge length. Tensile tests were carried out using a universal tensile testing machine (Instron 4208, 30-ton capacity, Norwood, MA, USA) at room temperature at a crosshead speed of 0.02 mm/s.

## 3. Results and Discussion

### 3.1. Visual and Macrostructure Examination

**Figure 3:**
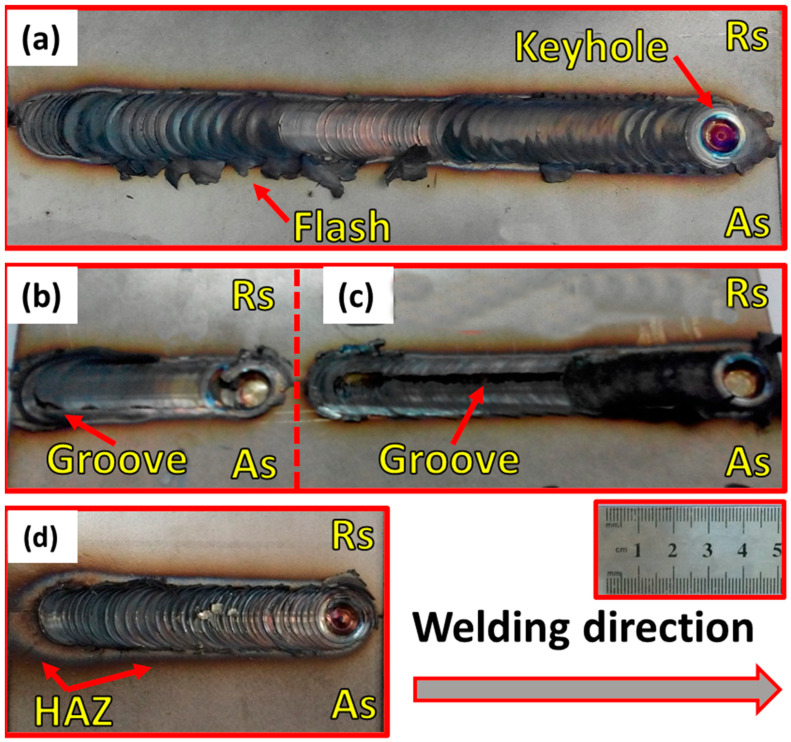
Top view appearance of 2205 DSS FSWed butt joints at welding parameters of (**a**) 600 rpm, 50 mm/min, and 14 kN, (**b**) 300 rpm, 50 mm/min, and 14 kN, (**c**) 300 rpm, 25 mm/min, and 14 kN, and (**d**) 300 rpm, 25 mm/min, and 20 kN.

**Figure 4:**
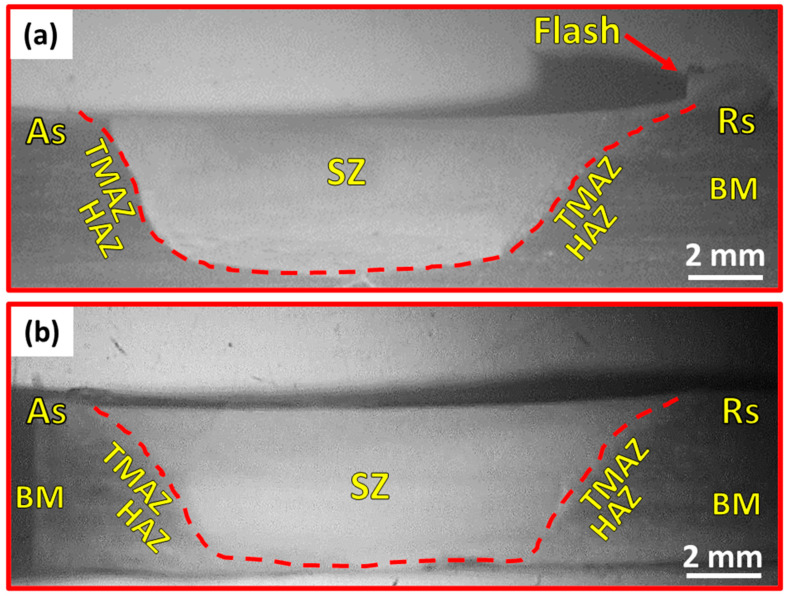
Macrostructures of transverse cross-sections of 2205 DSS butt joints FSWed at (**a**) 600 rpm, 50 mm/min, and 14 kN, and (**b**) 300 rpm, 25 mm/min, and 20 kN.

The top-view appearance of DSS 2205 FSWed butt joints at various welding parameters are illustrated in Figure 3. It can be observed that a surface groove defect-free butt joint with remarked flash is obtained at the FSW condition of 600 rpm, 50 mm/min, and 14 kN (Figure 3a). On the other hand, the welding condition of 300 rpm, 25 mm/min, and 20 kN (Figure 3d) produces an accepted surface defect-free butt joint. Furthermore, a remark groove-like defect is observed on the top surface at the advancing side for the 2205 DSS butt joint produced at the welding condition of 300 rpm, 50 mm/min, and 14 kN (Figure 3b), and also for the butt joint welded at 300 rpm, 25 mm/min, and 14 kN (Figure 3c). This groove-like defect can surely degrade the tensile properties of the weld joint. Some works [1,32] reported that groove-like defects are primarily generated with insufficient heat input during the stirring process. In this condition, the plastic material could not easily flow in the SZ to build the sound butt joint by the tool pin. In both numerical and experimental results, Fashami et al. [33] reported that the groove-like defect is formed in friction stir processing of AZ91 due to lower shoulder pressure and improper rotational and travel speeds of welding. In comparison, Zandsalimi et al. [34] ascribed the formed groove-like defect in the dissimilar joints between 430 stainless steel and AA6061 aluminium alloy to the high heat input that produces a plasticized material in the stir zone that cannot be kept by the tool shoulder. In the current study, the presence of the detected groove-like defect is likely to be a result of the insufficient downward force of 14 kN at a constant rotational speed of 300 rpm with the two travel speeds of 25 and 50 mm/min. One of the key welding process parameters that have an essential effect on the heat input is the tool rotational speed, travel speed, and downward force. Table 4 summarizes the visual inspection results of the 2205 DSS butt joints welded at different conditions. From Table 4, it can be concluded that the welding conditions of a rotational speed of 300 rpm and a downward force of 14 kN at both travel speeds of 25 and 50 mm/min failed to FSW of 5 mm 2205 DSS in defect-free butt joints. Thus, only the two visually accepted joints (Specimens S1 and S4) are subjected to macro and microstructures, hardness, and tensile property investigations. Figure 4 shows the macrostructures of transverse cross-sections of the FSWed 2205 DSS butt welds at different welding conditions. It can be observed that defect-free welds are gained at the welding conditions of a rotational speed of 600 rpm, a travel speed of 50 mm/min, and a downward force of 14 kN, as given in Figure 4a, and that butt joints are welded at 300 rpm, 25 mm/min, and 20 kN, as seen in Figure 4b.

### 3.2. Microstructure Examination

**Figure 5:**
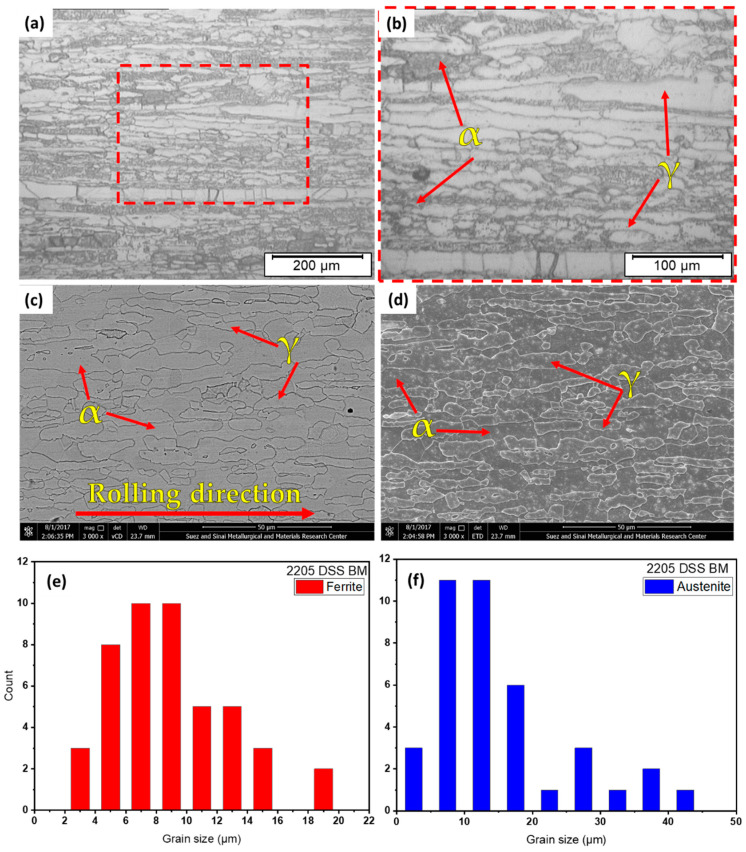
(**a**,**b**) Different magnifications of optical micrographs of the 2205 DSS BM. SEM images of the BM in two modes are given in (**c**) vCD and (**d**) ETD. (**e**,**f**) illustrate the BM grain size histograms for both α and γ, respectively.

The optical micrographs at different magnifications of the as-received 2205 DSS BM are illustrated in Figure 5a,b. Moreover, Figure 5c,d reveal the SEM micrographs of 2205 DSS using two types of detectors—a low k-volt high-contract detector (vCD) ((FEI Company, Hillsboro, OR, USA.) equipped with an AMETEK energy-dispersive spectroscope (EDS)) and an Everhart–Thornley detector (ETD) ((FEI Company, Hillsboro, OR, USA) equipped with an AMETEK energy-dispersive spectroscope (EDS)—to recognize the features of developed microstructures. The 2205 DSS BM has a typical microstructure of a typical wrought DSS consisting of austenite (γ) islands embedded in a ferrite matrix (α), as given in OM and SEM micrographs in Figure 5. It can also be remarked that both γ and α phases are elongated shapes in the rolling direction. The plotted grain size histograms of α and γ phases were performed using SEM analysis data and are presented in Figure 5e,f. The ferrite grain size ranges from 3 µm up to 19 µm with an average grain size of 8.80 µm, while the austenite grain size ranges from 2 µm up to 42 µm with an average grain size of 13.30 µm. This indicates the coarser grains of the austenite phase than the ferrite phase observed in the optical and SEM micrographs of the same figure.

**Figure 6:**
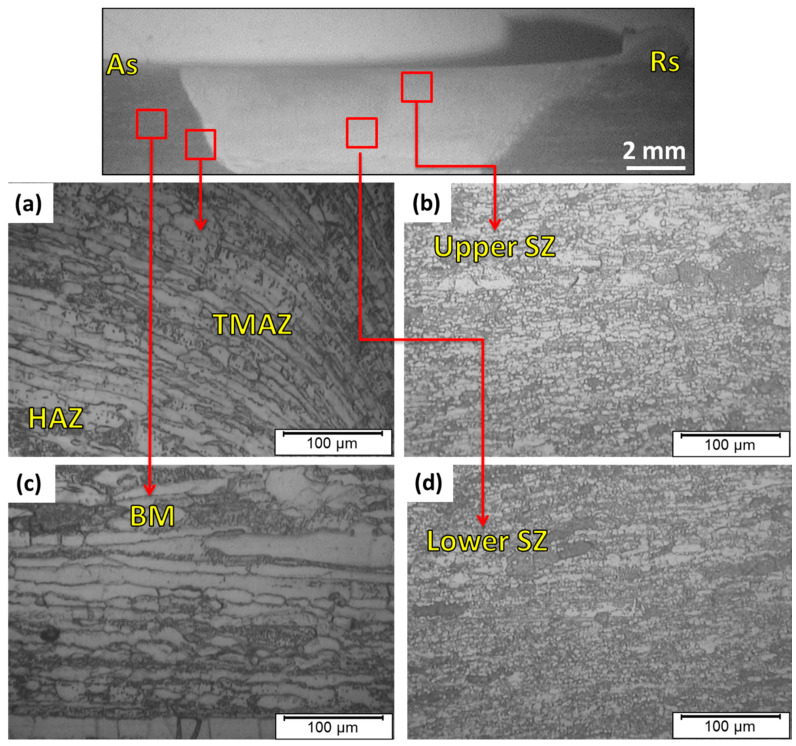
(**a**) Macrograph and (**b**–**d**) microstructure images of the different zones for FSWed butt joint of 2205 DSS welded at 600 rpm, 50 mm/min, and 14 kN.

**Figure 7:**
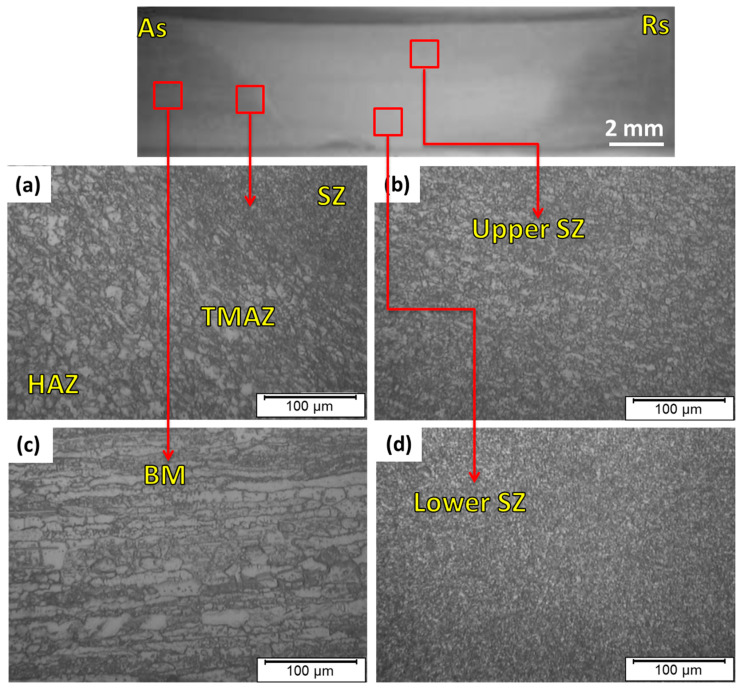
(**a**) Macrograph and (**b**–**d**) microstructure images of the different zones for the FSWed butt joint of 2205 DSS welded at 300 rpm, 25 mm/min, and 20 kN.

During the FSW process, three distinct zones are formed and surrounded by the BM. These three zones are the heat-affected zone (HAZ), the TMAZ, and the SZ. The SZ experiences the maximum temperature and plastic deformation. Moreover, the microstructure variation, in terms of grain size in the SZ, is significant relative to the surrounding zones: the TMAZ, the HAZ, and the BM. Figure 6 shows the optical micrographs of the detected SZ, TMAZ, and HAZ for the 2205 DSS butt joint welded at 600 rpm, 50 mm/min, and 14 kN. Furthermore, Figure 7 illustrates the micrographs of the same zones detected for the welded 2205 DSS butt joints at the processing parameters of 300 rpm, 25 mm/min, and 20 kN. For both welding conditions, the SZ consists of fine equiaxed grains in the upper and lower stir zones, as observed in Figure 6b,d and Figure 7b,d compared to the BM (Figure 6c and Figure 7a). The equiaxed refined grains are ascribed to the dynamic recrystallization process in the SZ [35,36,37,38]. In the TMAZ, a distorted structure due to the mechanical deformation and the heat effect is remarked in Figure 6a and Figure 7a. It can be noted that the grain size structure in the SZ in the welded joint at 300 rpm, 25 mm/min, and 20 kN are more refined than that remarked at the welding parameters of 600 rpm, 50 mm/min, and 14 kN.

**Figure 8:**
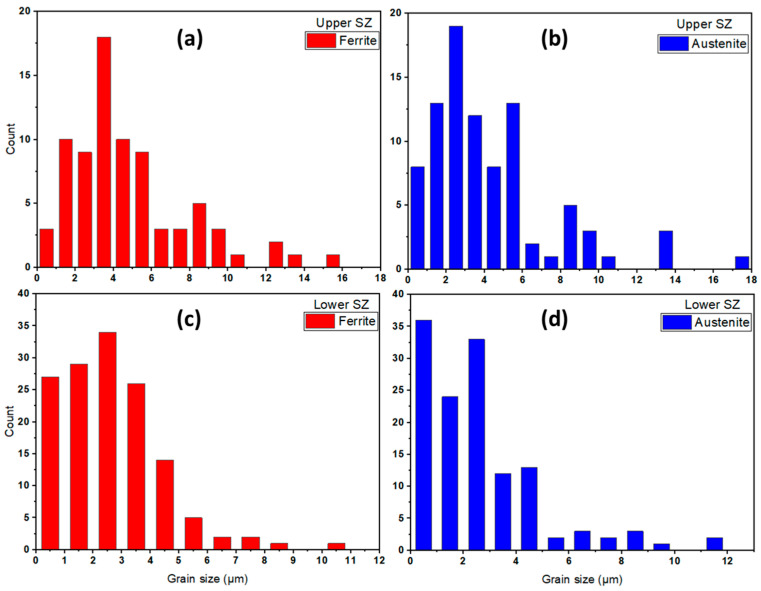
(**a**) Ferrite and (**b**) austenite grain size distribution histograms of upper zone, and (**c**) ferrite and (**d**) austenite grain size distribution histograms for 2205 DSS butt joint welded at 600 rpm, 50 mm/min, and 14 kN.

**Figure 9:**
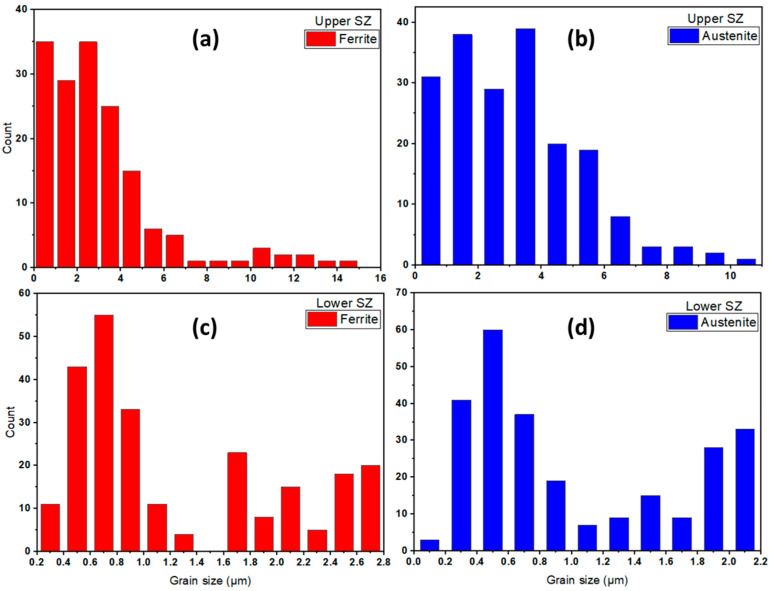
(**a**) Ferrite and (**b**) austenite grain size distribution histograms of the upper zone, and (**c**) ferrite and (**d**) austenite grain size distribution histograms for 2205 DSS butt joint welded at 300 rpm, 25 mm/min, and 20 kN.

Moreover, there is a slight difference in the α and γ grain size of the upper and lower zones of the SZ for both the welded joints, as detected during the SEM microstructure investigation. Figure 8 and Figure 9 represent grain size histograms of α and γ phases in the upper and lower stir zones of the FSW joints welded at the condition of 600 rpm, 50 mm/min, and 14 kN and at 300 rpm, 25 mm/min, and 20 kN, respectively. It can be seen from Figure 8 that the average grain size in the upper SZ is higher than that shown in the lower SZ for both α and γ phases. The average grain size values in upper SZ of α and γ are 4.80 ± 0.15 and 4.35 ± 0.19 µm, as given in Figure 8a,b, respectively. While, these values in the lower SZ are 4.11 ± 0.12 and 3.85 ± 0.15 µm, as shown in Figure 8c,d, respectively. For the joint welded at 300 rpm, 25 mm/min, and 20 kN, the average grain size values in upper SZ of α and γ are 2.93 ± 0.11 and 2.45 ± 0.13 µm, respectively, as given in Figure 9a,b. While, these values in the lower SZ are 2.51 ± 0.10 and 2.13 ± 0.12 µm, respectively, as given in Figure 9c,d. In the SZ, the material under the stirring process suffers from severe plastic deformation and frictional heat that causes dynamic recrystallization. The newly formed refined grains may suffer from the accumulated heat under the tool shoulder, causing grain growth in the upper SZ. Whereas, the grains far away from the shoulder toward the lower SZ may be subjected to fast cooling through the backing plate and keep the same grain size. Furthermore, in both welding conditions, the γ-phase displays more grain refining than the α-phase grains in the upper and lower regions of the SZ. Similar findings are reported by other authors for FSW of DSS [7,39].

### 3.3. Ferrite Content

The ferrite content of the as-received duplex stainless steel 2205 and its welds was measured using the instrument according to DIN EN ISO 17,655 standards. The obtained results are tabulated in Table 5. It can be noted that the present phases α and γ in the BM are 50.4% and 49.6%, respectively. These values are close to that reported by other authors for the as-received DSS 2205 [1,7]. In addition, from Table 5, the α: γ phase ratio for the welded butt joint at 600 rpm, 50 mm, and 14 kN is 49.4:50.6, while the joint welded at 300 rpm, 25 mm/min, and 20 kN has a ratio of 48.0:52.0. These balance ratios are around the balance ratio of α and γ for the BM. It was reported that the welded joint can be considered a high-quality joint if the γ phase percentage exceeds 30% in the 2205 DSS welded joint [40]. Under the welding parameters in the current study, the 2205 DSS were FSWed in the α and γ phases. The ferritization had not happened during the FSW process, and the slight range fluctuation of the α:γ ratio is likely to be a result of the original change in the ratio of the phases in DSS BM [29]. This result recommends FSW to weld DSS 2205 without changing in α and γ ratio at the suggested welding parameters in the current work.

### 3.4. Hardness and Tensile Properties

**Figure 10:**
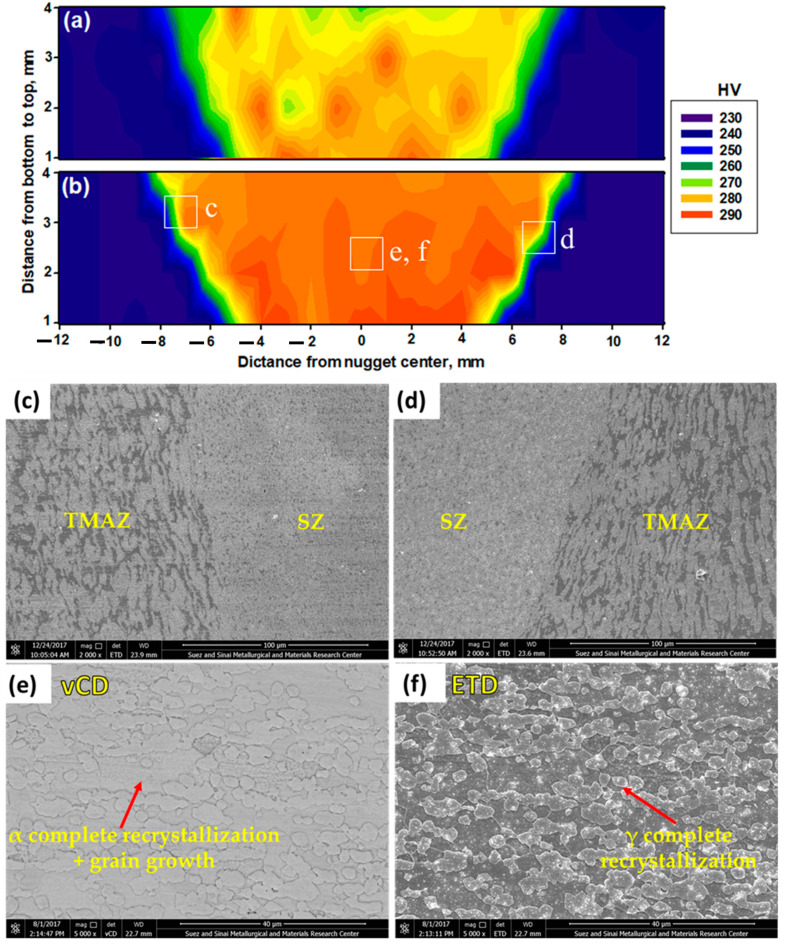
Hardness maps across through the transverse cross-section of the joints welded at (**a**) 600 rpm, 50 mm/min, and 14 kN, and (**b**) 300 rpm, 25 mm/min, and 20 kN. SEM images at the SZ–TMAZ interface (**c**) AS side and (**d**) RS side. SEM images of SZ for butt joint welded at 300 rpm, 25 mm/min, and 20 kN in (**e**) vCD and (**f**) ETD modes.

Figure 10 shows the hardness maps through the cross-sections of the 2205 DSS butt joints welded at 600 rpm, 50 mm/min, and 14 kN (Figure 10a), and at 300 rpm, 25 mm/min, 20 kN (Figure 10b). For both welds, it is noted that there is a remarked increase in hardness of the weld zone (SZ, TMAZ, and HAZ) compared to the 2205 DSS BM. The average hardness values of SZ, TMAZ, and HAZ are 273.2 ± 1.4 HV, 264 ± 1.5 HV, and 250 ± 1.9 HV, respectively, for the specimen welded at 600 rpm, 50 mm/min, and 14 kN. While, the hardness attains the values of 285.9 ± 1.6 HV, 274.2 ± 1.8 HV, and 264 ± 2.0 HV for the SZ, TMAZ, and HAZ for the specimen welded at 300 rpm, 25 mm/min, and 20 kN, respectively, compared to the hardness of BM (235 ± 3 HV). This hardness improvement in the SZ is related to the finer microstructure obtained compared to the grain structure of the BM, as seen in Figure 6 and Figure 7. The thermomechanical process in terms of severe plastic deformation [41,42] and maximum temperature in the SZ during the FSW [15,25] is responsible for the most effective microstructure features in terms of grain refining due to dynamic recrystallization. This justifies the highest hardness values obtained in the SZ region, as seen in both Figure 6b,d and Figure 7b,d. In the TMAZ, the lower temperature and plastic deformation over the SZ are established during the FSW process, leading to less grain refinement and lower average hardness values. Furthermore, in the HAZ, deformation is found, and the DSS only undergoes the influence of the thermal cycle [39]. These validate the lowest obtained hardness values close to the 2205 DSS BM in the HAZ regions. SEM is capable of achieving much higher magnification and resolution than OM. Thus, SEM was used for an in-depth examination of the microstructure features in the weld zone of the joint welded at 300 rpm, 25 mm/min, and 20 kN, which achieves the highest weld zone hardness, as seen in Figure 10b. Figure 10c,d illustrate the SEM images at SZ/TMAZ interfaces of the AS and RS, respectively. Figure 10e,f show the SEM images of the SZ in two modes, vCD, and ETD, respectively. The thermomechanical-affected zones in AS (Figure 10c) and RS (Figure 10d) showed a characteristic microstructure, in which the material (deformed γ the islands on an α matrix) flow in lines. The TMAZ has nearly the same feature microstructure as detected in both AS and RS. In the case of the DSS, both γ and α phases deform differently and recrystallize according to distinct kinetics [43,44]. The SZ (Figure 10) shows incomplete recrystallization of γ-phase, while the α-phase displays complete recrystallization and grain growth. Although the γ-phase has a higher recrystallization potential, the diffusion in the α-phase is quicker than the austenite one. Thus, the α-phase has recrystallization and grain growth fully, while the γ-phase shows only partial recrystallization. These results are in agreement with that reported by other authors [1,45]. Secondary phase precipitates were not observed in the SZ of the welded DSS 2205, as examined by the SEM vCD and ETD modes, seen in Figure 10e,f, respectively. This may probably be due to the shorter heating time [46].

The tensile properties of the welded joints depend on the joint efficiency and are governed by the welding parameters in terms of rotational speed, travel speed, and applied downward force. In addition, the failure during tensile tests occurred at the weakest part of the tested specimen. Tensile tests for the weld butt joints were performed at room temperature to measure the joint strength of the welded specimens compared to the BM. The yield strength (YS), ultimate tensile strength (UTS), elongation (E%), and failure location were recorded for each tested specimen. Moreover, the fracture surfaces were also investigated.

**Figure 11:**
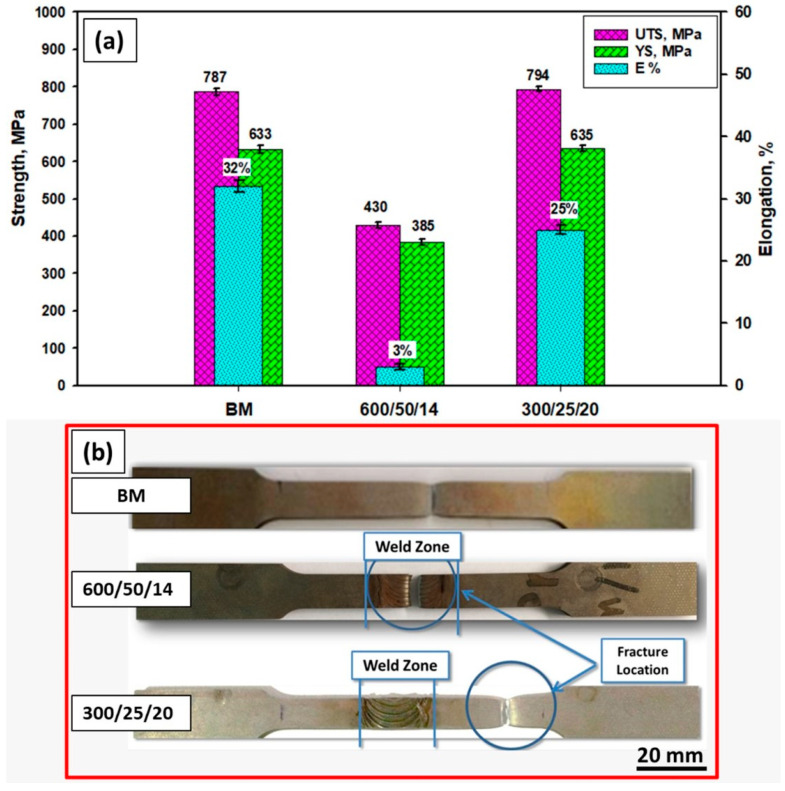
(**a**) Tensile properties of the 2205 DSS BM and the friction stir welds at different conditions and (**b**) failure location for the tensile tested specimens.

The tensile properties in terms of the UTS and Ys for the specimen welded at 600 rpm, 50 mm/min, and 14 kN showed significantly lower tensile properties relative to that of the BM as the sample failed in the NG zone. Failer inside the NG can occur due to the presence of a micro tunnel defect that can represent crack initiation and can propagate quickly with the tensile loading. On the other hand, the tensile properties of the butt joint welded at 300 rpm, 25 mm/min, and 20 kN are almost similar to the BM as the tensile sample failed in the base material zone. However, the elongation is slightly reduced from 32% for the BM to 24% after FSW. This result is confirmed with the hardness measurements shown in Figure 11a,b. This improvement in UTS and YS is mainly attributed to the developed microstructure modification through the FSW [47]. Ghadar et al. [48] concluded that friction stir processing 2205 DSS promotes significant UTS and ductility enhancement compared to as-received material. Moreover, they ascribed this enhancement to grain size refining. The grain size in the weld zone is a dominant parameter governing mechanical properties. It was observed that the fracture location of the butt joint FSWed at 600 rpm, 50 mm/min, and 14 kN occurred in the SZ, as shown in Figure 11b. While, the fracture location of the specimen welded at 300 rpm, 25 mm/min, and 20 kN has occurred faraway the weld zone (in BM), indicating higher joint efficiency than the other weld specimen and the BM, as seen in Figure 11b.

**Figure 12:**
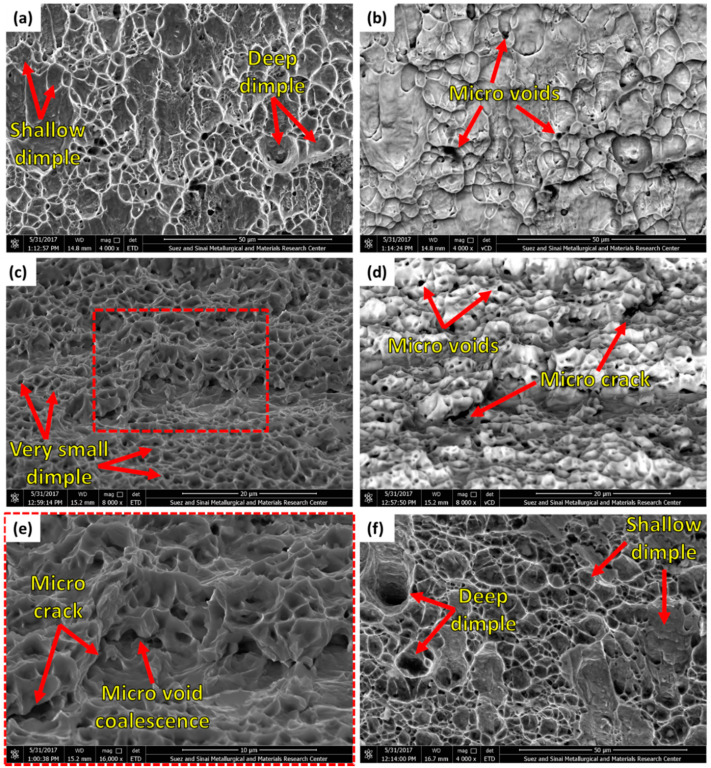
SEM micrographs for the fracture surfaces of (**a**) BM in ETD and (**b**) vCD modes; (**c**) welded joint at 600 rpm, 50 mm/min, and 14 kN, fractured at the SZ in ETD; (**d**) vCD modes; (**e**) higher magnification of (**c**); and (**f**) welded joint at 300 rpm, 25 mm/min, and 20 kN, fractured at the BM in the ETD mode.

Figure 12 shows the SEM fractography of the fracture surfaces of the 2205 DSS BM and the fractured welded joints, which generally indicates characteristics of ductile fractures. The fracture surface of BM (Figure 12a,b) shows shallow and deep dimples in different sizes and shapes (elongated and rounded) related to the as-received microstructure, which consists only of α and γ phases. The presence of the two phases can be confirmed as there are no second phase particles observed inside the dimples, as given in Figure 12b. Furthermore, microvoids are detected in the fractured surface of the BM. The butt joint welded at 600 rpm, 50 mm/min, and 14 kN failed at the SZ (Figure 11b). The fracture surface in Figure 12c–e shows very small equiaxed dimples related to grain size present in the SZ (Figure 7b,d).

Moreover, microvoids and micro cracks are detected in the fracture surfaces. These features indicate ductile fracture mode, and the fracture mechanism is a microvoids coalescence (Figure 12e). Whereas, the butt joint produced at 300 rpm, 25 mm/min, and 20 kN failed at the BM (the softest section compared to the weld zone (Figure 11b). The fracture surface features (Figure 12f) are the same as the fractured BM (Figure 12a).

## 4. Conclusions

The effect of different FSW processing parameters, i.e., rotational speed, travel speed, and downward force, on the microstructure and mechanical properties of the FSWed 2205 DSS was investigated, and, based on the gained results, the following conclusions can be mentioned:

1. The designed WC welding tool of the high pin to shoulder ratio (65%) was successfully used several times to conduct FSW of 5 mm thick DSS 2205 and produced defect-free butt joints. This design can be suggested to extend the WC tool life.
2. The elongated ferrite matrix and austenite islands in the DSS 2205 BM were significantly refined after FSW in the stir zone and the average grain size of the ferrite and austenite reduced from 8.8 µm and 13.3 µm for the base material to 2.71 µm and 2.24 µm, respectively.
3. The average hardness values of the SZ, TMAZ, and HAZ attained 273.2 HV, 264 HV, and 250 HV, respectively, for the butt joint welded at 600 rpm, 50 mm/min, and 14 kN. While, they reach the values of 285.9 HV, 274.2 HV, and 264 HV, respectively, for the joint welded at 300 rpm, 25 mm/min, and 20 kN, compared to the hardness of BM (235 HV).
4. The butt joint welded at 600 rpm, 50 mm/min, and 14 kN failed at the SZ with significantly lower tensile properties, whereas the joint welded at 300 rpm, 25 mm/min, and 20 kN failed at the BM, indicating high joint quality, with almost similar tensile properties to that of the BM.

## Figures and Tables

**Table 1:** The 2205 DSS Chemical Composition (in wt.%).

C	Mn	Si	Cr	Ni	Mo	P	S	V	Cu	Fe
0.030	0.838	0.001	21.500	5.290	3.660	0.024	0.001	0.132	0.093	Balance

**Table 2:** FSW Parameters that were used to Weld the 2205 DSS Butt Joints.

Sample Code	Rotational Speed (rpm)	Travel Speed (mm/min)	Downward Force (kN)
S1	600	50	14
S2	300	50	14
S3	300	25	14
S4	300	25	20

**Table 3:** Dimensions of the FSW Tool Shoulder and Pin used in this Work.

Shoulder diameter	20 mm
Shoulder feature	concave shoulder
Pin length	4.5 mm
Pin profile	Tapered cylindrical
Pin diameter	Pin base: 13 mm
	Pin tip: 9 mm
Tilt angle	3°

**Table 4:** Visual Inspections for the FSWed 2205 DSS Butt Welds.

Specimen No.	Figure	Visual Appearance	Surface Defect		Test Results
			Flash	Groove	
S1	Figure 3a	Good	much	Non	Accepted
S2	Figure 3b	Partially good	Small	Detected	Rejected
S3	Figure 3c	Bad	Small	Detected	Rejected
S4	Figure 3d	Very good	Very small	Non	Accepted

**Table 5:** Ferrite Number Readings (%) for the 2205 DSS BM and the SZ of the Butt Welded Joints.

Sample	FSW Conditions (rpm)/(mm/min)/(kN)	Ferrite Number Readings (%)					AV of Measured α Phase	Calculated γ Phase
BM	-	50	49	51	50	52	50.4	49.6
S1-SZ	600/50/14	53	46	49	49	50	49.4	50.6
S4-SZ	300/25/20	48	46	48	51	47	48.0	52.0

## Data Availability

The data presented in this study are available on request from the corresponding author. The data are not publicly available due to the extremely large size.
